# Genetic Ancestry-Smoking Interactions and Lung Function in African Americans: A Cohort Study

**DOI:** 10.1371/journal.pone.0039541

**Published:** 2012-06-21

**Authors:** Melinda C. Aldrich, Rajesh Kumar, Laura A. Colangelo, L. Keoki Williams, Saunak Sen, Stephen B. Kritchevsky, Bernd Meibohm, Joshua Galanter, Donglei Hu, Christopher R. Gignoux, Yongmei Liu, Tamara B. Harris, Elad Ziv, Joseph Zmuda, Melissa Garcia, Tennille S. Leak, Marilyn G. Foreman, Lewis J. Smith, Myriam Fornage, Kiang Liu, Esteban G. Burchard, for the Health ABC and CARDIA Studies

**Affiliations:** 1 Division of Epidemiology, Department of Thoracic Surgery, Vanderbilt University, Nashville, Tennessee, United States of America; 2 Departments of Medicine and Bioengineering and Therapeutic Sciences, University of California San Francisco, San Francisco, California, United States of America; 3 Children's Memorial Hospital, Department of Pediatrics, Northwestern University Feinberg School of Medicine, Chicago, Illinois, United States of America; 4 Department of Preventive Medicine, Northwestern University Feinberg School of Medicine, Chicago, Illinois, United States of America; 5 Center for Health Policy and Health Services Research and Department of Internal Medicine, Henry Ford Health System, Detroit, Michigan, United States of America; 6 Department of Epidemiology and Biostatistics, University of California San Francisco, San Francisco, California, United States of America; 7 Sticht Center on Aging, Wake Forest University School of Medicine, Winston-Salem, North Carolina, United States of America; 8 College of Pharmacy, University of Tennessee Health Science Center, Memphis, Tennessee, United States of America; 9 Department of Epidemiology and Prevention, Wake Forest University, Winston-Salem, North Carolina, United States of America; 10 Laboratory of Epidemiology, Demography and Biometry, Intramural Research Program, National Institute on Aging, Bethesda, Maryland, United States of America; 11 Department of Epidemiology, Graduate School of Public Health, University of Pittsburgh, Pittsburgh, Pennsylvania, United States of America; 12 Division of Pulmonary and Critical Care Medicine, Morehouse School of Medicine, Atlanta, Georgia, United States of America; 13 Division of Pulmonary and Critical Care, Department of Medicine, Northwestern University Feinberg School of Medicine, Chicago, Illinois, United States of America; 14 Institute of Molecular Medicine and Division of Epidemiology, School of Public Health, University of Texas Health Sciences Center, Houston, Texas, United States of America; The University of Texas M. D. Anderson Cancer Center, United States of America

## Abstract

**Background:**

Smoking tobacco reduces lung function. African Americans have both lower lung function and decreased metabolism of tobacco smoke compared to European Americans. African ancestry is also associated with lower pulmonary function in African Americans. We aimed to determine whether African ancestry modifies the association between smoking and lung function and its rate of decline in African Americans.

**Methodology/Principal Findings:**

We evaluated a prospective ongoing cohort of 1,281 African Americans participating in the Health, Aging, and Body Composition (Health ABC) Study initiated in 1997. We also examined an ongoing prospective cohort initiated in 1985 of 1,223 African Americans in the Coronary Artery Disease in Young Adults (CARDIA) Study. Pulmonary function and tobacco smoking exposure were measured at baseline and repeatedly over the follow-up period. Individual genetic ancestry proportions were estimated using ancestry informative markers selected to distinguish European and West African ancestry. African Americans with a high proportion of African ancestry had lower baseline forced expiratory volume in one second (FEV_1_) per pack-year of smoking (−5.7 ml FEV_1_/ smoking pack-year) compared with smokers with lower African ancestry (−4.6 ml in FEV_1_/ smoking pack-year) (interaction *P* value  = 0.17). Longitudinal analyses revealed a suggestive interaction between smoking, and African ancestry on the rate of FEV_1_ decline in Health ABC and independently replicated in CARDIA.

**Conclusions/Significance:**

African American individuals with a high proportion of African ancestry are at greater risk for losing lung function while smoking.

## Introduction

Tobacco smoking is the leading cause of preventable deaths in the United States and is an important contributor to accelerated lung function decline [1]. While European American individuals tend to smoke greater numbers of cigarettes when compared with African American individuals [2], [3], African Americans appear to have greater nicotine intake from tobacco smoke and lower renal clearance of cotinine [4]. In addition, African Americans have lower normative lung function when compared with European Americans [5], [6] and African ancestry is associated with lower pulmonary function [7]. Despite these known disparities in smoking patterns, pharmacokinetics, and lung function, investigations of smoking-related diseases have been conducted primarily in European populations with relatively few studies devoted to other population groups. Therefore, the extent to which differences in exposure and susceptibility to tobacco smoke impact racial differences in lung function decline is unclear [8], [9]. This question may have clinical or public health relevance since African American individuals may be particularly susceptible to smoking related diseases such as chronic obstructive lung disease [8], [9], [10], [11], [12].

Strong evidence from recent genome-wide association studies conducted in European American individuals implicates a genetic component to reduced lung function [13], [14], [15], [16], [17]. Additionally, family-based studies, including a study conducted in adult African Americans [18], suggest that there is a strong heritable component to lung function [19]. These heritability estimates increase among smokers [17], suggesting that the decline in lung function due to smoking is also genetically determined. Given the described differences by population, we hypothesized that the effect of tobacco smoking on lung function differs by genetic ancestry (i.e., a measure of one's African and European heritage). In the present study, we examined African Americans which have, on average, a mixed ancestry of approximately 80% African ancestry and 20% European ancestry [20]. We investigated interactions between African genetic ancestry and tobacco smoking on forced expiratory volume in one second (FEV_1_) and its rate of decline in a large population of African Americans.

## Methods

### Ethics statement

All participants gave written informed consent. The Institutional Review Boards at the University of Pittsburgh, the University of Tennessee and the University of California, San Francisco approved the Health ABC protocol. The CARDIA study is reviewed annually by the Institutional Review Boards at the University of Alabama Birmingham, Northwestern University Feinberg School of Medicine, University of Minnesota and Kaiser Permanente in Oakland. CARDIA participants sign a new informed consent form at every examination.

### Health ABC Study Population

Self-identified European American (N = 1,794) and African American participants (N = 1,281) were enrolled into the ongoing prospective Health, Aging and Body Composition (Health ABC) cohort study between April 1997 and June 1998. Subjects were eligible for participation if they were Medicare recipients, aged 70–79 years, and resided near Pittsburgh, Pennsylvania or Memphis, Tennessee. Additional eligibility criteria for the Health ABC Study participants included speaking English, being free from a life-threatening illness, and willingness to remain in the study area for ≥3 years. Persons were excluded if they met any of the following criteria: a) difficulty with daily living activities, defined as any difficulty walking a quarter of a mile or any difficulty walking up 10 steps without resting; b) cognitive impairment; c) inability to communicate with an interviewer; or d) treated for cancer within the previous three years. Further details of the Health ABC study population have been published elsewhere [21], [22].

### Interview Data Collection and Pulmonary Function Assessment

Demographic data and detailed smoking histories were collected from Health ABC participants using a structured questionnaire during an extensive home interview conducted within year one of study enrollment. Smaller versions of the questionnaire were implemented during subsequent years of the study. Individuals who reported smoking <100 cigarettes were categorized as never smokers and individuals who reported smoking at least 100 cigarettes during their lifetime (i.e. five or more packs) were queried about smoking initiation, cessation and average cigarettes per day. Using this information we categorized participants as current smokers and former smokers and estimated the number of smoking pack-years (i.e., [no. cigarettes smoked per day*no. of years smoked]/20).

Methods employed for assessment of pulmonary function in Health ABC study participants are described elsewhere in greater detail [21]. Briefly, three to five spirometry tracings were obtained from enrolled participants during years 1, 5, 8 and 10 of the study period. All spirometry results were reviewed and scored for acceptability and reproducibility by two study staff. Pulmonary function tests meeting American Thoracic Society (ATS) criteria for acceptability and reproducibility were included in statistical analyses. This analysis includes participants who self-identified as African American and whose pulmonary function testing met the ATS standards.

### Genotyping and Estimation of Genetic Admixture in Health ABC

Health ABC participants provided a blood sample from which genomic DNA was extracted and whole genome amplified. A panel of 1,536 autosomal biallelic ancestry informative markers was selected for genotyping in Health ABC African Americans. Markers were selected to distinguish the continental ancestral populations comprising African Americans [23], [24]. The ancestry informative marker (AIM) panel was developed by selecting SNPs across the genome with high informativeness for ancestry between European and African continental populations. Genetic ancestry informative markers for West African and European ancestry were genotyped using Illumina technology. Methods for genotyping of SNPs informative for genetic ancestry, including the quality checks applied, have been previously described [23], [24]. AIMs genotyping data were also available from West African (N = 104) and European (N = 241) ancestral populations for improvement of individual admixture estimation. For estimation of individual ancestry we included only AIMs found to be in Hardy Weinberg Equilibrium (HWE) and in linkage equilibrium among the West African and European ancestral populations [24]. Genetic ancestry of the African American participants was determined using a final set of 1,332 AIMs and the software program STRUCTURE [25] to estimate the proportion of African and European ancestry in the Health ABC African Americans, assuming two ancestral populations.

### Independent Replication of Longitudinal Analyses Coronary Artery Disease in Young Adults (CARDIA) Study

The CARDIA Study is a prospective cohort initiated in 1985, with 5,115 participants enrolled between the ages of 18 to 30 years from four clinical sites. Of these, 2,637 self-identified as African American. Detailed study design, recruitment procedures, and baseline findings have been previously reported [26], [27]. Written informed consent was obtained for all subjects and IRB approval was obtained from each participating institution. For this analysis we included all non-asthmatic African American subjects with AIMs genotyping data available (N = 1,223). Spirometry was measured during years 0, 2, 5, 10, and 20 in accordance with ATS criteria for accuracy and precision [28]. Subject's data was only included after age 25 to allow for lung function decline estimation starting from an age at which plateau lung function is typically attained. Smoking status was determined by standardized questionnaire at each visit and individuals were classified as never, former, or current smokers. Individual African ancestry in the African American study participants was determined using an independent panel of 42 biallelic autosomal markers having large allele frequency differences (delta >0.3) between African and European populations [29]. As demonstrated by other investigators, this number of AIMs is sufficient for estimating ancestry among African Americans [30]. The software program STRUCTURE [25] was used to estimate individual admixture proportions using a K = 2 estimate for the number of ancestral subpopulations.

### Statistical Analysis

Linear regression models were used to estimate the effect of African ancestry, coded as a continuous variable, on the association between tobacco smoke exposure and baseline FEV_1_ among African Americans without a self-reported diagnosis of pulmonary disease. Selection of covariates included in regression models was done using *a priori* knowledge of variables impacting pulmonary function resulting in the selection of age, sex, height, family income, and clinic site as covariates. Continuous variables were mean centered using measures collected at the time of study enrollment. Baseline analyses removed observations with standardized residuals greater than 2.5 (N = 5, indicating few statistical outliers). Two-way interaction terms between percent African ancestry and smoking pack-years were included in full linear regression models to estimate p-values for interactions. Linear regression models were also stratified by median African ancestry (80.8%) or smoking status (ever versus never) to assess interactions.

Linear mixed effects models examined the influence of African ancestry on tobacco smoke exposure and FEV_1_ decline, adjusting for the same covariates as in baseline analyses. Restricted maximum likelihood estimation was implemented to examine the impact of African ancestry on the association between tobacco smoke exposure and FEV_1_ decline while simultaneously accounting for the correlation structure between repeated pulmonary measures over the 10-year follow-up period in Health ABC. Smoking status was allowed to vary over time and continuous measures were mean centered. Similar linear mixed effects models were evaluated in CARDIA examining the relationship between African ancestry and smoking status (never, current, and former) on rate of pulmonary function decline. Covariates included sex, clinic, height, height-squared, body mass index (BMI), smoking pack-years and maximum attained education. Three-way interaction terms between age (as a measure of time), percent African ancestry, and smoking status were included in mixed models to estimate p-values for interactions. A two-sided significance probability of 0.05 was used to infer presence or absence of interaction. A p-value of 0.2 indicated suggestive evidence for the presence or absence of interaction, as a recommended strategy to reduce type II error and avoid missing a true interaction [31] and successfully implemented by others [32], [33]. Analyses were performed using SAS, version 9.2 (Cary, North Carolina).

## Results

In its initial year, 1,281 African Americans, mean age 73.9 years, were enrolled in the Health ABC study. Repeat measures of pulmonary function were obtained during years one, five, eight and ten. At year one 942 African Americans had acceptable spirometry results and during years five, eight and ten there were 587, 406, and 337 individuals, respectively, had acceptable spirometry results; the remaining study individuals were either lost to follow-up or deceased over the ten year follow-up period (see Figure S1). Only 160 individuals (12%) reported having had a physician diagnosis of asthma, chronic obstructive pulmonary disease, chronic bronchitis, or emphysema at the initial visit and 77 (48%) of these 160 reported using pulmonary medications. The majority of African Americans in Health ABC had at least a high school education (56%) and 49% had a low income ($10,000–$25,000). Most African American participants were never smokers (45%), 39% were former smokers and 16% were current smokers. Mean smoking pack-years was 30 pack-years with an average of 12 cigarettes smoked per day (Table 1).

**Table 1.** Baseline demographic characteristics of 1,281 African Americans participating in the Health ABC study.

Characteristic		N (%)
Age, mean years (SD)		73.9 (2.9)
Male sex (%)		552 (43.0)
Education, years (%)		
	< High school	556 (43.6)
	High school graduate	389 (30.5)
	> High school	330 (25.9)
	Unknown	6
Income, $ (%)		
	<10,000	296 (26.2)
	10,000–25,000	553 (48.9)
	25,001–49,999	225 (19.9)
	≥ 50,000	56 (5.0)
	Unknown	151
Smoking status (%)		
	Never smokers	573 (44.8)
	Current smokers	207 (16.2)
	Former smokers	498 (39.0)
	Unknown	3
Smoking pack-years†, mean (SD)		30.3 (25.4)
Age initiated smoking, years, mean (SD)		19.6 (7.0)
Age stopped smoking, years, mean (SD)		51.0 (14.0)
Average cigarettes smoked per day‡, mean (SD)		12.4 (8.6)
Pulmonary function, mean (SD)		
	FEV_1_ (milliliters)	1972.2 (575.7)
	FVC (milliliters)	2551.8 (724.9)
% African ancestry, mean (SD)		78.5 (13.1)
	Unknown	126

Definition of abbreviations: BMI  =  body mass index; FEV_1_  =  forced expiratory volume at one second; FVC  =  forced vital capacity.

Categorical and continuous variables were assessed with chi-square test and two-sample t-test, respectively.

† among ever smokers only.

‡ among current smokers only.

### Baseline cross-sectional analyses

A total of 813 African Americans had both acceptable quality spirometry data and individual genetic ancestry estimates. Multivariable linear regression analyses demonstrated an inverse relationship between FEV_1_ and both African ancestry and amount smoked. Smoking was associated with a lower FEV_1_ among individuals with both high African ancestry (defined as greater than median value of 80.8%) and those with low African ancestry (less than or equal to 80.8%). The reduction in baseline FEV_1_ associated with smoking was greater among those with higher African ancestry. Table 2 illustrates that among smokers only African Americans with high African ancestry had lower baseline FEV_1_ per pack-year smoked (−5.7 ml FEV_1_/ smoking pack-year, 95% confidence interval [CI]: -8.1, -3.3) compared to individuals with low African ancestry (−4.6 ml in FEV_1_/ smoking pack-year, 95% CI: -7.5, -1.6). This difference was considered suggestive of an interaction between African ancestry and tobacco smoking pack-years exposure (*P* for interaction  = 0.17) on baseline FEV_1_. Stratifying by smoking status (ever vs. never) also revealed a greater reduction in lung function with increasing African ancestry among smokers compared to never smokers (−4.4 ml in FEV_1_ versus −1.4 ml in FEV_1_), although the interaction between African ancestry and smoking status was not statistically significant (Table 3). We also examined FEV_1_/FVC and did not observe a significant interaction between African ancestry and smoking (data not shown).

**Table 2:** Baseline associations between FEV_1_ and smoking, stratified by African ancestry, among smokers with quality spirometry and no pulmonary disease in the Health ABC study.

	<80.8% African ancestry			≥80.8% African ancestry		
	(N = 169)			(N = 196)		
Variable	Beta	SE	*P* value	Beta	SE	*P* value
Intercept	2121.67	165.37	<0.001	2126.33	121.53	<0.001
Age, years	−14.36	12.16	0.24	−14.56	9.66	0.13
Female	−383.46	100.53	<0.001	−375.70	80.60	<0.001
Smoking, pack-years	−4.58	1.50	0.003	−5.69	1.21	<0.001
Standing height, mm	18.72	5.18	<0.001	21.29	4.32	<0.001
Household income	57.59	41.77	0.17	9.71	36.71	0.79
Pittsburgh	47.11	72.90	0.52	64.66	57.87	0.27

Definition of abbreviations: SE  =  standard error; mm  =  millimeter.

Observations with standardized residuals > 2.5 were removed. Median African ancestry is 80.8%, estimated from 1332 ancestry informative markers.

*P* value for interaction for percent African ancestry*smoking pack-years = 0.17, estimated from full model.

**Table 3:** Baseline associations between FEV_1_ and African ancestry, stratified by smoking status, among African Americans with quality spirometry and no pulmonary disease in the Health ABC study.

	Never smokers			Ever smokers		
	(N = 270)			(N = 371)		
Variable	Beta	SE	*P* value	Beta	SE	*P* value
Intercept	2081.05	212.02	<0.001	2354.20	190.92	<0.001
% African ancestry†	−1.43	1.92	0.46	−4.40	1.80	0.02
Age, years	−19.62	7.85	0.01	−13.44	7.75	0.08
Female	−233.20	63.96	<0.001	−340.92	63.98	<0.001
Standing height, mm	24.12	3.51	<0.001	19.21	3.37	<0.001
Household income	35.19	30.49	0.25	30.46	28.21	0.28
Pittsburgh	95.33	48.29	0.05	30.62	46.15	0.51

Definition of abbreviations: SE  =  standard error; mm  =  millimeter.

Observations with standardized residuals >2.5 were removed.

*P* value for interaction for percent African ancestry*smoking status = 0.38, estimated from full model.

† African ancestry is coded as a continuous variable.

### Longitudinal analyses

**Figure 1:**
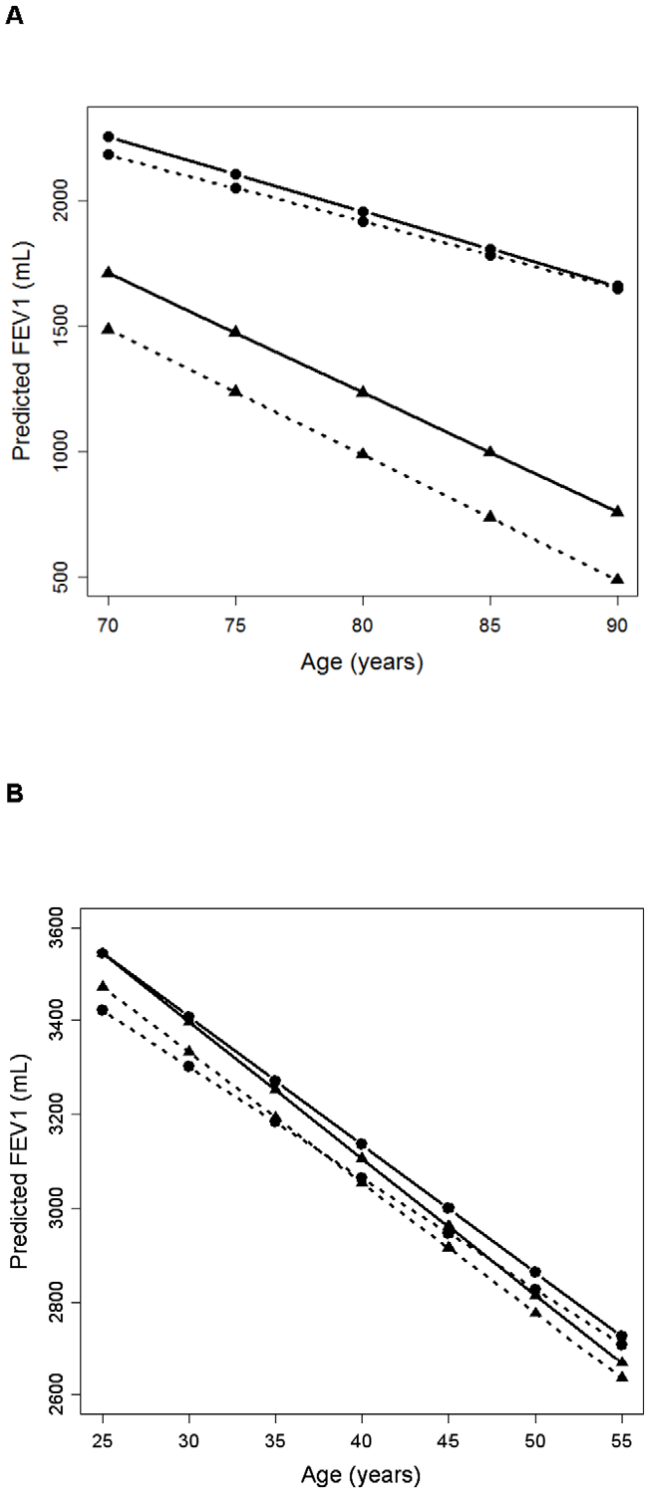
Predicted FEV_1_ decline (in milliliters) by smoking-ancestry strata derived from a mixed effects model. Health ABC (A) and CARDIA (B). Current smokers are represented by the ▴ symbol and former smokers by the • symbol. Low African ancestry groups are represented by a solid line and high African ancestry by a dashed line. The groups are as follows: low African current smokers (solid line with ▴ symbol), high African current smokers (dashed line with ▴ symbol), low African former smokers (solid line with • symbol), and high African former smokers (dashed line with • symbol).

Multivariable linear mixed effects models examining the rate of decline in FEV_1_ revealed a three-way interaction suggestive of a complex relationship between age, smoking, and individual African ancestry in the Health ABC study population, although results did not meet statistical significance (Table 4: Model 1 *P* = 0.14 for smoking pack-years; Model 2 *P* = 0.16 for former smokers). A sensitivity analysis removed individuals reporting pulmonary disease at baseline and found the effect was in the same direction although no longer statistically significant (*P* value >0.20). We applied a similar mixed effects model for FEV_1_ to the CARDIA study population (see Table S1). The numbers and ages of participants included at each visit are described in Table S2. We observed a suggestive interaction between age, former smoking status, and African ancestry (*P* = 0.12) among CARDIA participants (Table S3) even though this population was significantly younger than Health ABC participants. The longitudinal rate of decline in FEV_1_ in both Health ABC and CARDIA is illustrated in Figure 1 (Figure 1A: Health ABC, Figure 1B: CARDIA). While current smokers with high African ancestry had lower lung function than current smokers with low African ancestry, the rates of decline were similar between these two groups. Former smokers with high African ancestry also had lower lung function at baseline than former smokers with low African ancestry. However, in the case of former smokers, the rate of decline was less in individuals with high African ancestry. Among those who quit smoking, participants with higher African ancestry had a slower lung function decline, such that after follow-up, former smokers had similar lung function regardless of their proportion of African ancestry. These trends were observed in both Health ABC and CARDIA participants. We also examined FEV_1_/FVC in Health ABC and found a significant interaction between former smoking status, African ancestry and age (*P* = 0.02) and a suggestive interaction between smoking pack-years, African ancestry and age (*P* = 0.12).

**Table 4:** Longitudinal association between FEV_1_ rate of decline, smoking and African ancestry, among African American participants in the Health ABC study.

	FEV_1_ (ml/yr)		
	(N = 1,737)		
Variable	Beta	SE	*P* value
*Model 1* †			
Intercept	1630.22	58.43	<0.001
African ancestry, %	−2.90	1.40	0.04
Age, year at time t	−26.45	10.18	0.01
Smoking, pack-years	−4.60	0.79	<0.001
Age*African*Pack-years	0.007	0.004	0.14
*Model 2*			
Intercept	1629.17	58.66	<0.001
African ancestry, %	−4.37	1.70	0.01
Age, year at time t	−31.55	2.24	<0.001
Smoking, pack-years	−4.61	0.77	<0.001
Age*African*Former smoker	0.32	0.23	0.16
Age*African*Current smoker	0.12	0.43	0.78

Mixed effects models are adjusted for sex, standing height, household income, clinic site and two-way interactions among covariates. Age, height and African ancestry are mean centered.

† Model 1 is also adjusted for smoking status.

## Discussion

Using two independent adult cohorts, we demonstrated that smoking has a greater impact on reduced lung function among African Americans with high African ancestry than those with low African ancestry. Importantly, we found smokers with high African ancestry had lower baseline lung function than individuals with lower African ancestry. Inclusion of the 1,332 AIMs separately in models examining the association between tobacco smoke exposure and baseline FEV_1_ did not abrogate the response, even after correcting for multiple testing using false discovery rate [34], suggesting none of the ancestry informative markers were responsible for the observed associations. Moreover, baseline examination of FEV_1_ among European Americans indicated that their smoking-associated reduction in lung function was less (−3.9ml FEV_1_/ smoking pack-year) than in African Americans. It is well recognized that smoking leads to reduced lung function and increases the rate of lung function decline with increasing age [35]. Despite this knowledge and established racial/ethnic differences for pulmonary function, few studies have examined the complex relationship between race, smoking, and rate of lung function decline among healthy adults. Using genetic ancestry, we can elucidate racial/ethnic differences in smoking-related lung function decline. Genetic ancestry itself is unlikely the cause of these differences, but may be a proxy for population-specific rare genetic variants contributing to racial/ethnic differences in lung function and susceptibility to tobacco smoke [36]. Novel approaches, such as admixture mapping, may enable us to identify population-specific variants that contribute to lung function decline [37], [38].

Prior to our study, other groups have found inconsistent associations between race and smoking associated lung function decline. Vollmer *et* *al*. examined racial differences in smoking-related effects on cross-sectional FEV_1_ in European American and African American individuals and found no statistically significant difference in smoking associated decline [39]. This is distinct from the findings of others who identified a greater susceptibility to FEV_1_ decline per pack-year smoked and a greater susceptibility to COPD among African American individuals [10], [11], [40]. Some of the inconsistencies in prior investigations of racial differences in smoking-related pulmonary function may be due to their cross-sectional design [39] or designs which matched on smoking [12], thereby precluding the ability to examine its impact on racial differences in pulmonary measures. With statistical approaches only recently developed for estimating admixture, prior studies were unable to quantify the degree of African ancestry. Our study compliments and extends prior investigations comparing African American and European American individuals by examining smoking-related changes in lung function in relation to a continuous measure of African ancestry.

Our findings suggest that tobacco-related lung damage and African ancestry together have a substantially greater impact on lung function and perhaps its decline than either factor alone. In other words, the relationship between smoking and pulmonary function depends on the level of African ancestry. Individuals with high African ancestry are particularly susceptible to the impact of cigarette smoking on the decline of FEV_1_. In contrast to current smokers, former smokers with high African ancestry have lung function that becomes more similar to individuals with low African ancestry, suggesting lung function improves with smoking cessation irrespective of African ancestry. In fact, former smokers with high African ancestry may have an even greater benefit from smoking cessation, such that their lung function approaches that of former smokers with low African ancestry. These findings reveal greater complexity in the relationship between race and tobacco smoking associated lung function decline than was seen in an earlier study [9]. Our findings suggest lung function depends on smoking status and the proportion of individual African ancestry and highlight the importance of smoking cessation. Future studies with greater statistical power are needed to confirm this relationship.

To assess potential survival bias, we compared baseline characteristics between those lost to follow-up and individuals remaining in the Health ABC study. Individuals remaining in the study were more likely to be male, higher BMI, higher education, fewer smoking pack-years, and lower African ancestry (Table S4) compared to those lost to follow up during the study period suggesting survival bias may impact our findings. However, we found similar associations in the younger CARDIA cohort with a 20-year follow-up and 65% retention for African American participants, thus supporting the robustness of our findings. CARDIA participants included in analyses were slightly more educated and included more females than participants not included in analyses, but were similar for other key demographic characteristics (Table S5). The consistency of the associations showing greater effect sizes in individuals of higher African ancestry for various measures of smoking suggests that smoking and African ancestry together may have an important impact on pulmonary function decline over time. We recognize that this observation may be due to social, environmental and genetic factors that co-vary with genetic ancestry. For example, environmental factors which could co-vary with ancestry include discrimination and proximity to roadways. The reduction in lung function in those with greatest African ancestry may have occurred in the distant past due to childhood or adolescent exposure to tobacco smoke leading to suboptimal lung growth or may be due to rapid decline in lung function in the early years of smoking.

Health ABC provides a robust approach for observing baseline spirometry measures, but also allows for assessment of longitudinal changes in lung function associated with smoking in this unique population of older African American adults. Replication of our findings in the CARDIA African American participants provides additional support. Few studies have ascertained repeated pulmonary function measures in either older populations or African American individuals. Thus the collection of repeated pulmonary function measures over a ten year time period in Health ABC and over 20 years in CARDIA is a notable strength of this study. Additionally, stringent protocols were implemented to ensure that quality spirometry measures were obtained. The prospective design of CARDIA and Health ABC minimizes possible participant response bias in reporting of smoking exposures.

In summary, we have demonstrated that African ancestry and tobacco use act synergistically to accelerate rate of lung function decline. Specifically, our results suggest that individuals with high African ancestry have increased risk of smoking-related changes in lung function and may be an important group for smoking intervention. In addition, the observation that former smokers with high African ancestry may have a less rapid rate of decline in lung function suggests that this group may preferentially benefit from cessation. Our findings have important public health implications. We have demonstrated that genetic ancestry may serve as a biomarker for identifying smokers who would benefit from targeted counseling regarding smoking cessation [41], [42]. One important implication of our findings is that there may be rare genetic variants relevant to smoking associated lung function decline that are population-specific and which co-vary with genetic ancestry [43]. While we cannot rule out that some of these associations may be in part due to environmental factors which co-vary with ancestry, these results highlight the scientific advantages of studying racially mixed populations. Future analyses should include admixture mapping to identify genomic regions associated with rate of lung function decline. These regions are likely to harbor rare population-specific variants that could illuminate pathogenetic pathways involved in the regulation of tobacco metabolism and lung function [36]. Reduced lung function is a predictor of overall morbidity and mortality [44], [45]; therefore, it is imperative to recognize and intervene in the populations at greatest risk.

## Supporting Information

Figure S1 **Flow chart of African Americans participating in the Health ABC Study.**
(PDF)

Table S1 **Baseline demographic characteristics of 1,223 African Americans participating in the CARDIA study.**
(PDF)

Table S2 **Number and mean ages of participants in the CARDIA study.**
(PDF)

Table S3 **Longitudinal association between FEV1 decline, tobacco smoking and African ancestry, among African Americans participating in the CARDIA study.**
(PDF)

Table S4 **Demographic characteristics of Health ABC African Americans lost to follow-up versus participants remaining in the study through year 10.**
(PDF)

Table S5 **Demographic characteristics of non-asthmatic CARDIA African Americans who were included or not included in study sample due to lack of genetic data.**
(PDF)
