# Supplementary figures and images for: Genetic Ancestry-Smoking Interactions and Lung Function in African Americans: A Cohort Study
Source: PLoS One. 2012 Jun 21;7(6):e39541. doi: 10.1371/journal.pone.0039541 (PMC3380861; doi:10.1371/journal.pone.0039541)

**Figure S1. Flow chart of African Americans participating in the Health ABC Study.**

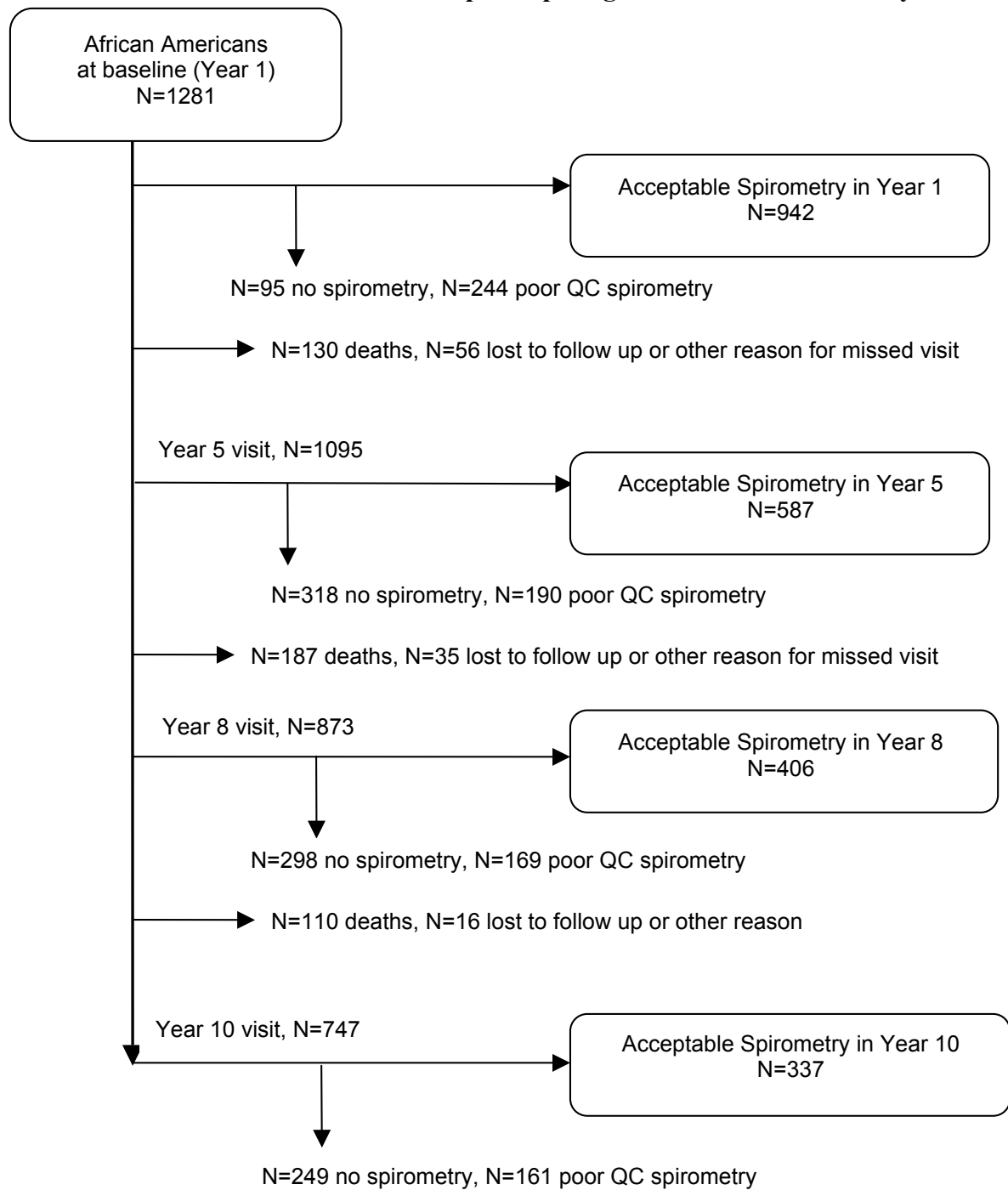

Supplement: Figure S1 — Flow chart of African Americans participating in the Health ABC Study. (PDF) [file pone.0039541.s001.pdf]
