# Supplementary material for: Genetic Ancestry-Smoking Interactions and Lung Function in African Americans: A Cohort Study
Source: PLoS One. 2012 Jun 21;7(6):e39541. doi: 10.1371/journal.pone.0039541 (PMC3380861; doi:10.1371/journal.pone.0039541)
Supplement: Table S1 — Baseline demographic characteristics of 1,223 African Americans participating in the CARDIA study. (PDF) [file pone.0039541.s002.pdf]

Table S1. Baseline demographic characteristics of 1,223 African Americans participating in the CARDIA study.

Characteristic	N=1,223<br>N (%)
Age, mean years (SD)	24.4 (3.8)
Male sex (%)	535 (43.7)
Education, years	
< High school	52 (4.3)
High school graduate	257 (21.0)
> High school	914 (74.7)
Smoking status	
Never smokers	737 (60.7)
Current smokers	384 (31.6)
Former smokers	94 (7.7)
Unknown	8
Smoking pack-years*, mean (SD)	4.1 (4.4)
Average cigarettes smoked per day†, mean (SD)	10.6 (6.5)
Pulmonary function, mean (SD)	
FEV_1 (milliliters)	3319.1 (673.5)
FVC (milliliters)	3956.0 (869.3)
% African ancestry, mean (SD)	70.5 (12.7)

Definition of abbreviations: FEV_1 = forced expiratory volume at one second; FVC = forced vital capacity.

*among ever smokers only

†among current smokers only
