# Supplementary material for: Genetic Ancestry-Smoking Interactions and Lung Function in African Americans: A Cohort Study
Source: PLoS One. 2012 Jun 21;7(6):e39541. doi: 10.1371/journal.pone.0039541 (PMC3380861; doi:10.1371/journal.pone.0039541)
Supplement: Table S2 — Number and mean ages of participants in the CARDIA study. (PDF) [file pone.0039541.s003.pdf]

Table S2. Number and mean ages of participants in the CARDIA study.

Exam year	N	Mean age (SD), year
0	576	27.7 (1.8)
2	760	28.9 (2.5)
5	951	30.4 (3.2)
10	1163	34.4 (3.8)
20	889	44.6 (3.8)

All subjects included were over age 25 and had pulmonary function data available at each examination year
