# Supplementary material for: Genetic Ancestry-Smoking Interactions and Lung Function in African Americans: A Cohort Study
Source: PLoS One. 2012 Jun 21;7(6):e39541. doi: 10.1371/journal.pone.0039541 (PMC3380861; doi:10.1371/journal.pone.0039541)
Supplement: Table S3 — Longitudinal association between FEV1 decline, tobacco smoking and African ancestry, among African Americans participating in the CARDIA study. (PDF) [file pone.0039541.s004.pdf]

Table S3. Longitudinal association between FEV_1 decline, tobacco smoking and African ancestry, among African Americans participating in the CARDIA study.

	FEV_1 (ml/yr) (N=1221)		
Variable	Beta	SE	p-value
Intercept	3106.76	28.16	< 0.0001
African ancestry, %	-6.71	1.19	< 0.0001
Age, year at time t	-26.75	0.84	< 0.0001
Smoking, pack-years	-4.54	1.86	0.01
Past Smoking	-18.71	24.70	0.45
Current Smoking	-7.41	19.77	0.71
Age*African*Former smoker	0.18	0.12	0.12
Age*African*Current smoker	0.03	0.11	0.78

*Linear mixed effects models are adjusted for sex, standing height, height squared, maximum achieved education, BMI, clinic site and two-way interactions between ancestry and age as well as between age and smoking status. Age, height and African ancestry are mean centered.
