# Supplementary material for: Genetic Ancestry-Smoking Interactions and Lung Function in African Americans: A Cohort Study
Source: PLoS One. 2012 Jun 21;7(6):e39541. doi: 10.1371/journal.pone.0039541 (PMC3380861; doi:10.1371/journal.pone.0039541)
Supplement: Table S4 — Demographic characteristics of Health ABC African Americans lost to follow-up versus participants remaining in the study through year 10. (PDF) [file pone.0039541.s005.pdf]

**Table S4. Demographic characteristics of Health ABC African Americans lost to follow-up versus participants remaining in the study through year 10.**

| Characteristic | Lost to follow-up† (N=687) n (%) | Remaining in study (N=594) n (%) | *P* value |
|---|---|---|---|
| Male sex | 351 (51.1) | 201 (33.8) | < 0.01 |
| BMI (kilograms/meters$^2$) (SD) | 28.2 (5.4) | 29.0 (5.4) | < 0.01 |
| Education, years | | | < 0.01 |
| < High school | 344 (50.4) | 212 (35.8) | |
| High school graduate | 194 (28.5) | 195 (32.9) | |
| > High school | 144 (31.4) | 186 (31.4) | |
| Unknown | 6 | | |
| Income, $ | | | 0.05 |
| <10,000 | 171 (28.1) | 125 (24.0) | |
| 10,000 – 25,000 | 304 (49.9) | 249 (47.8) | |
| 25,001 – 49,999 | 111 (18.2) | 114 (21.9) | |
| ≥ 50,000 | 23 (3.8) | 33 (6.3) | |
| Unknown | 151 | | |
| Smoking pack-years‡, mean (SD) | 20.3 (26.4) | 12.3 (20.4) | < 0.01 |
| Pulmonary function, mean (SD) | | | |
| $FEV_1$ (milliliters) | 1898.8 (612.5) | 1959.2 (530.1) | 0.07 |
| FVC (milliliters) | 2556.2 (759.6) | 2546.8 (684.5) | 0.82 |
| % African ancestry, mean (SD) | 79.4 (12.8) | 77.5 (13.4) | 0.01 |
| Unknown | 126 | | |

Definition of abbreviations: BMI = body mass index; $FEV_1$ = forced expiratory volume at one second; FVC = forced vital capacity.

*Categorical and continuous variables were assessed with chi-square test and two-sample t-test, respectively.

†Lost to follow-up indicates either missing (N=70) or died (617).

‡among ever smokers only
