# Supplementary material for: Genetic Ancestry-Smoking Interactions and Lung Function in African Americans: A Cohort Study
Source: PLoS One. 2012 Jun 21;7(6):e39541. doi: 10.1371/journal.pone.0039541 (PMC3380861; doi:10.1371/journal.pone.0039541)
Supplement: Table S5 — Demographic characteristics of non-asthmatic CARDIA African Americans who were included or not included in study sample due to lack of genetic data. (PDF) [file pone.0039541.s006.pdf]

Table S5. Demographic characteristics of non-asthmatic CARDIA African Americans who were included or not included in study sample due to lack of genetic data.

Characteristic	Not included (N=830) n (%)	Included (N=1223) n (%)	*P* value
Year 0 age, mean (SD)	24.1 (3.8)	24.4 (3.8)	0.17
Male sex	404 (48.7)	535 (43.7)	0.03
BMI (kilograms/meters^2) (SD)	24.9 (5.4)	25.3 (5.7)	0.17
Maximum attained education, years, mean (SD)	13.8 (3.6)	14.6 (4.1)	< 0.001
Smoking pack-years at Year 0, mean (SD)	1.8 (3.5)	1.7 (3.4)	0.30
Smoking pack-years at Year 0*, mean (SD)	4.2 (4.1)	4.3 (4.4)	0.67
Pulmonary function, mean (SD)			
FEV_1 (milliliters)	3322.5 (704.0)	3319.1 (673.5)	0.91
FVC (milliliters)	3960.7 (871.7)	3956.0 (869.3)	0.91

BMI denotes body mass index; FEV_1, forced expiratory volume at one second; and FVC, forced vital capacity. Categorical and continuous variables were assessed with chi-square test and two-sample t-test, respectively.

*among current smokers only
